# On the Diseases of Old Age, and Their Cure

**Published:** 1844-01

**Authors:** 


### ART. V.

*Die Krankheiten des höheren Alters und ihre Heilung dargestellt* von Dr. C. CANSTATT, &c.—*Erlangen*, 1839.

*On the Diseases of Old Age, and their Cure.* By Dr. C. CANSTATT, Royal Bavarian Medical Jurist, &c.—*Erlangen*, 1839. Two Vols. 8vo, pp. 268, 422.

FEW English writers have treated on the hygiene of old age, and fewer still on its diseases. Welsted's 'Liber de Ætate vergenti,' and Floyer's "Gerocomica," both published in 1724, are among the earliest. Half a century later, Benjamin Rush wrote an essay on the state of the mind and body in old age, and some remarks on the diseases of aged persons; and in 1838, Sir Anthony Carlisle gave his experience on the diseases of childhood and old age to the world in his 'Practical Observations on the Preservation of Health," &c. Fifty octavo pages only of his small volume are devoted to the hygiene of old age. Foreign literature has been much more fruitful in works on the subject; but many of them are merely small pamphlets, others are adapted mainly to the popular reader, and the greater number are monographs on some special diseases of old age. A book comprising in one comprehensive plan the information diffused through these varied publications and through the works on general pathology must, therefore, be considered a desideratum for the physician. Such is Dr. Canstatt's publication, with the additional recommendation that the special branch of pathology treated of is brought into relation with the newest physiology and general pathology. We have read the work carefully, and we can give it our unqualified approbation. Some little faults excused, it is a model of composition. The subject-matter is well arranged; the details are accurate; the style of writing classical; the description of particular diseases clear and vivid. That there is *no* room for criticism could not be affirmed of any book; but we would say, there is so little here that we shall make our review almost entirely analytical.

Old age (*senectus*) is the period of natural decline. Plants and animals describe an imaginary semicircle. They commence from a vital point, are gradually evolved until they attain their climax, and then they as gradually decline to death. The segment of the circle from the climacteric point to death is old age, or, as our author terms it, the *Involution-period*, as the antagonistic segment is the *Evolution-period.* This involution-period is characterized, physiologically, by a return of the organism to the commencement of the evolution-period, although its direct course is to its mother-earth. On this hypothesis, Dr. Canstatt occasionally founds a comparison between the physiology and pathology of childhood and old age, and a demonstration of their identity at many points. We need not say that these views are more curious than useful. Fortunately, we do not find the pathology of our author infected with them; they are confined entirely to his physiological views.

At what age does the period of decline commence? Hippocrates fixed it at seven times seven years, or forty-nine; and its termination, or old age, at ten times seven, or seventy years. Riverius subdivided it into three minor periods: 1, *senectus prima*, extending from the fiftieth to the sixtieth year; 2, *ætas ingravescens*, from the sixtieth to the seventieth; and 3, *decrepitudo*, or the days of labour and sorrow mentioned by the psalmist, and which fill up the interval between the age of seventy and the grave. Experience, however, plainly teaches that the periods of decline cannot be measured with anything like accuracy. The poetical declaration of sacred writ, that "wisdom is gray hairs to a man, and an unspotted life is old age," is often verified in a very matter-of-fact manner. A green old age, a *senectus prima*, at three score and ten, is often the reward of temperance and moderation in living, while decrepitude overtakes the moderately-intemperate at fifty, and death itself comes on but a little later. The involution of special organs cannot be taken as an index of the general involution. Muscular power often fails long before the intellectual, and *vice versâ:* often an old man of seventy, who has long been toothless, may be seen walking about as actively and as upright as a man of forty. The cessation of menstruation in females indicates commencing involution; but we can only apply this analogically to males, for the power of procreation is possessed by the latter to an advanced period of life. Our author, making the involution-period and old age conterminous, leaves it to the practitioner to ascertain the latter by determining the limits of the former in each individual case. And on this view is the limitation of his subject constituted. All diseases of old age are not necessarily diseases of the involution-period, for aged people are often equally exposed with the young to the causes of diseases in general. But these may be modified by the involution, and hence two pathological divisions of the subject, the one comprising those diseases necessarily connected with the physiological changes of decline, the second including those which are only modified by the physiological conditions of the involution-period.

*Anatomy and physiology of old age.* Dr. Canstatt remarks that a knowledge of these is essential to the right knowledge and treatment of disease. The unwary practitioner, for example, is often deceived by an apparent hardness or fulness of the pulse into actively depleting measures and a fatal destruction of the vital power, when the indication in question is dependent entirely upon hypertrophy or ossification of the radial artery. The general appearance of old people is well known. According to Quetelet, man arrives at his maximum weight at forty, and woman at fifty years of age. According to Fischer, the specific gravity of old people is diminished, so that poor old women might float when thrown into water, and yet be innocent of witchcraft. As the hair turns gray the skin becomes less moist, and more parchment-like, and all the cutaneous secretions are impeded. Hence the difficulty of establishing critical discharges through the skin, and the propriety of directing immediate attention in case of failure, to the more practicable organs—the kidneys and bowels. The contractility and vitality of the cellular tissue is diminished in the involution-period; the tissues generally are consequently less speedily and effectively renewed, effete parts less readily taken up by the absorbents, and morbid products more readily deposited from the slowly-circulating blood, thus predisposing to cancerous degenerations indurations, &c. The osseous system partakes in this vascular inactivity, and the controversy respecting the non-union of the neck of the femur, the olecranon, &c. in case of fracture, might easily have been avoided, as our author observes, by a reference to the physiology of old age. The diminished deposit of fat in the cellular tissue renders a warmer clothing necessary.

The changes in the vascular system are of importance with reference to the diseases of the involution-period. According to some writers, the heart is less, and its walls thinner; the muscular fibres small and flaccid, and portions here and there appear to have a membranous structure only. Passive aneurisms are frequent with softening or dilatation. Fat is deposited round the heart, and bony matter in its inner membrane and in the valves, giving origin to peculiar affections. The arteries are also said to be diminished in caliber; the smaller ones obliterated, and the larger partially ossified, or deprived of their gelatine: hence their liability to rupture. Baillie, Stevens, and Astley Cooper have remarked arterial ossification so frequently in old people, that they consider it almost the normal state. Bizot's recent researches in some degree have set aside these statements. This observer asserts, in contradiction to Béclard, that the walls of the left ventricle and the septum increase in thickness as age advances; in contradiction to Bichat, he found that the aorta was increased in caliber in old age, as indeed are all the arterial trunks. But the opinions of the English writers just mentioned are confirmed by Bizot's observations. In short, the two great changes in the arterial system are: 1, gradual increase in capacity, and hypertrophy of the cardiac structures; and 2, the deposit of a new substance in the middle tunic of the arteries which gradually destroys their fibres, and changes them into an atheromatous matter. Other alterations of structure are doubtless connected with the latter. Opacity of the membranous portion of the semi-lunar valves is very frequent after the age of thirty-nine. It was observed by Bizot in 80 females out of 100, aged from sixty to eighty-nine years, and in 92 men in 100 of the same age. Spots of ossification frequently appear in the same structures in females about the age of sixty-eight, in men at the age of forty-nine. The *corpora aurantii* are often cartilaginous and increased in size. The concretions on the aorta and arteries of old people are composed of crystals, according to Gluge. The last-mentioned observer found these crystals also on the surface of the heart. The veins rarely ossify; Bichat never witnessed this morbid change. They become dilated, sometimes in a remarkable degree, and varicose; especially in the liver and brain.

The change in the fibrous, muscular, and osseous structures of old age are well known. The tendons ossify, the bones are devoid of medulla, more brittle, and thinner and smaller; the muscular fibres waste, occasionally ossify, and are sometimes changed into a fatty substance; in which case, the bones are also softened. Gluge states that it is not the muscles themselves that are changed, but that drops or globules of fat are deposited among the primitive fibrils. The cartilages of the larynx are rarely found not ossified (more or less) after the age of fifty. The cartilaginous rings of the bronchial tubes ossify also, and the cartilages of the ribs. The process proceeds from the centre of the cartilage to the circumference. Fibrous cartilages rarely change; the ligaments of the spine are frequently found ossified; the cartilages scarcely ever. Bone may be thus united to bone, as the ilium to the sacrum, the *ossicula auditus* to each other, &c. The serous membranes are often found dry, thick, and opaque; and ossification of the arachnoid, of the lining of the pericardium, and of the serous membrane of the eye have been observed.

The changes in the nervous system are not less numerous and remarkable. Less water and more albumen enter into its composition. The brain and spinal cord are harder, yellowish, and dry; the gray substance diminishes; the neurilema is thicker and more fibrous. The brain and nerves diminish in size and weight. According to Desmoulins, the brain at the age of seventy is from a fifteenth to a twentieth lighter and smaller than at middle age.

Canstatt proceeds to details the state of the alimentary, respiratory, urinary, and sexual systems in old age at great length, as also those observed in the organs of the senses. Respiration, manducation, digestion, and assimilation, absorption, secretion, &c. are reviewed, together with the functions of the liver, spleen, and intestinal canal. In considering these various points, reference is constantly made to the most recent views: Schultz's, for example, (lately noticed by us,) are noticed here. The secondary pathological changes consequent upon the morbid are also illustrated. This second chapter closes with the following apt description of the "soul-life" of the old man, which we quote as a specimen of our author's style:

"The intellectual powers of the aged usually continue unimpaired longer than the physical. The experience of a long life has ripened the judgment; hence the wisdom of the old is proverbial. But this spiritual life is nourished rather from matter already stored up, than enlivened by fresh additions through the external senses. The functions which assimilate these to the inner self are wanting. The impressions made on the senses are imperfect, and lead the old man to form erroneous ideas and judgments with reference to the outer world; only his experience comes in to his aid and corrects his error. The imaginative powers of the old man are lost; he views the future, for the most part, as gloomy and sorrowful; he is serious, and little susceptible of joy. His memory fails, yet the recollection of the circumstances of his youth is fresh, and the general law seems to hold good, and in the aged soul, as in the aged body, those faculties which appear the earliest are active the longest. The aged man is observant, and thinks deeply on particular circumstances. He carries his affairs of business into the depths of his soul. Caution and profound thought take the place of early levity. The honour of a cautious and mature judgment belongs to the aged. His conclusions bear the stamp of deep thought and long experience. The fire of the passions and emotions burns dimly in him; he is now free from the storm which once roused an uproar in his soul. Conscious how transitory are both joy and sorrow, he looks calmly and self-collectedly on the circumstances amidst which his short life is prolonged. The present has no value for him; he lives only in the recollection of the past, and is an intolerant censor of the present, because he compares the shadows of age with the brightness of his youthful years. The old man easily becomes egotistical, mistrustful, superstitious, misanthropic, covetous, mean-spirited. Many old people are remarkable for irritability of temper and disposition, and for a morose and intolerant manner, so that they are a burden and a nuisance to all around them.

'Multa senem circumveniunt incommoda; vel quod
Quærit, et inventis miser abstinet, et timet uti,
Vel quod res omnes timide gelideque ministrat;
Dilator, spe longus, iners, avidusque futuri,
Difficilis, querulus, laudator temporis acti
Se puero, censor castigatorque minorum.'—Horace." (pp. 89-90.)

We pass the fourth chapter, on the climacteric periods, without remarks; from the fifth, on the etiology and hygiene of old age we shall make some excerpta.

Dr. Canstatt thinks that in the present age corporeal health and strength are offered up to the moloch of (so-called) civilization. The existing generation, he thinks, presents fewer examples of long life than the departed. Less civilized nations and individuals, whose occupations demand manual without intellectual labour, arrive at a greater age than nations and individuals with whom the contrary happens. The most numerous examples of longevity are, he asserts, to be found amongst the poor, laborious, and illiterate. This statement is, doubtless, positively true, but relatively false. It is positively true, because the poor and labouring classes are the more numerous. Dr. Canstatt does not bear in mind the important fact that high intellectual powers are often the result of weak corporal powers, and *vice versâ*, and that the controlling force of society is exerted upon those in whom the innate intellectual power predominates by virtue of which they are attracted to those pursuits requiring superior mental qualifications. Hence the apparent greater mortality of the class. It should be added, too, that the pursuits to which they are drawn necessarily place the individual in a state of intellectual warfare, and are accompanied by much mental anxiety—a condition of mind much more injurious to the vegetative system than any intellectual efforts. The physician, barrister, merchant, or artist, is much more exposed to this wear and tear of struggle and anxiety than the clergyman or university professor. These latter have not the common anxieties of life superadded, and as a stimulus to their mental activity. Blumenbach died at the age of eighty-eight; Lord Lynedoch is above ninety; the present Archbishop of York is an advanced octogenarian; the late Earl of Cathcart was eighty-eight when he died; Talleyrand was about the same age: the latter may, however, be an example of the power which hereditary longevity has of counteracting the wear of a stormy life. This hereditary longevity is one of the best established facts in physiology. There is scarcely a graveyard in the United Kingdom without illustrations of it.* And although as a general rule temperance

* We wish some one competent to the task would undertake to investigate hereditariness. The subject has never been considered in its totality, and would amply reward the *scientific* inquirer.

in youth is the surest guarantee for that old age which "as a lusty winter is frosty but kindly," yet there are many with the hereditary gift who have "applied hot and rebellious liquors to their blood," and yet walk erect at four score. The following are the marks of the constitution which predisposes to longevity:

"Such subjects grow slowly and regularly: the head is small, relatively, to the body; the forehead rough and covered with wrinkles; the neck neither long nor thin, nor swelled out; the complexion in youth not too florid. They have also sound closely-set teeth, which are sometimes reproduced in advanced life; a broad and deep chest; round full shoulders; a flat and contracted belly; strong torose extremities, thickly covered with stiff hair; a rough skin; and the hair of the head harsh, bristly, and rather blond than black. Early grayness, without baldness, is a mark of longevity according to Bacon. The respiration is easy, full, slow, regular, and scarcely observable; the voice strong; the pulse slow, strong, and not easily altered in rhythm. The cutaneous secretion must be free, but not profuse; the renal secretion small; the stools firm and infrequent; the sleep refreshing; appetite and digestion good; the mind rather inclined to gaiety than seriousness, and not easily disturbed by emotions." (p. 104.)

We have some excellent dietetic rules in this chapter. Old people ought to take food in small quantities, and never to repletion; for large quantities are not fully digested, and partly pass through the alimentary canal unchanged. A full stomach presses dangerously on the thoracic viscera and the larger blood-vessels. After eating, active exercise, whether of mind or body, should be avoided; and a *siesta* is generally recommended. Should, however, the face become red during sleep, and the individual awake with vertigo and a dull headach, it is better to avoid it. Milk is an excellent article of diet; well-fermented malt liquor may be taken, and also good old wines free from acidity and astringent matter. Old hocks or French wines and Alicant, Madeira, or Malaga, are the best. Tea and coffee in moderation are also suitable.

The temperature of the atmosphere has an important influence on the mortality of old people. Statistic researches have verified the dictum of Celsus, "Senes æstate et autumni primâ parte tutissimi." The following tables exhibit the fact:

*Part of a Table from Quetelet.**

| Ages. | Jan. | Feb. | Mar. | April | May. | June. | July. | Aug. | Sept. | Oct. | Nov. | Dec. |
|---|---|---|---|---|---|---|---|---|---|---|---|---|
| 50 to 65 ... ... | 1·30 | 1·22 | 1·11 | 1·02 | 0·93 | 0·85 | 0·77 | 0·85 | 0·89 | 0·90 | 1·00 | 1·15 |
| 65 to 75 ... ... | 1·43 | 1·32 | 1·18 | 0·99 | 0·91 | 0·77 | 0·71 | 0·80 | 0·88 | 0·86 | 0·98 | 1·17 |
| 75 to 90 ... ... | 1·47 | 1·39 | 1·16 | 1·01 | 0·87 | 0·77 | 0·67 | 0·75 | 0·84 | 0·84 | 1·00 | 1·21 |
| 90 and upwards ... | 1·58 | 1·48 | 1·25 | 0·96 | 0·87 | 0·75 | 0·64 | 0·66 | 0·76 | 0·74 | 1·03 | 1·29 |

*Deaths at Troyes for ten years;* 1821-30.†

| | | | | | | | | | | | | |
|---|---|---|---|---|---|---|---|---|---|---|---|---|
| Old people, at all ages ... | 0·83 | 0·74 | 0·76 | 0·76 | 0·54 | 0·47 | 0·39 | 0·39 | 0·40 | 0·42 | 0·62 | 0·63 |

Looking at these tables, the practitioner, we think, will not hesitate to recommend a warmer climate to his aged patients during the winter

* This comprises about 400,000 of different ages, and applies to all Belgium for the years 1827 to 1831.

† Patier, Lancette Franç. 1835, No. 107.

months, if circumstances permit; and if not, will take care to show the importance of a regulated temperature. Old people, we think, should never sleep in a room without a fire if the temperature be at or below 45° to 50° Fahr. The more provident amongst the ancient Romans betook themselves to Naples when they found old age creeping upon them.

Elderly people commit a grievous error when they utterly abandon their customary pursuits, and often with those their usual exercise of body and mind. Nothing is so injurious to health as this stagnant *otium cum dignitate*. It often lays the foundation both of bad habits and of bad health, and those lead to premature death, especially from apoplexy. But anxious cares are as injurious as idleness. Easy circumstances and placid pursuits promote longevity. When a man insures a sum of money on his life or an annuity, he so far removes a fertile source of mental anxiety and bodily disease, and proportionally assists his digestive and assimilative powers. He has, in fact, insured an addition of years. With a similar object in view there should be a gradual withdrawal from the cares of business, only retaining so much of the latter as will afford a healthful excitement. Our author recommends the society of children in walking, conversation, play, &c., as particularly suitable to the aged. Nature herself, in the penchant which decrepitude exhibits for infantile life, points to this.

Dr. Canstatt, in his fifth chapter, lays down general principles regarding the treatment of senile diseases, some of which we will notice. It is important to remember that old age is itself a state of disease. The actual standard of health is below par, and the efforts of the physician are injurious when directed to the attainment of anything beyond the maintenance of this state. The *vis medicatrix naturæ* has less scope for action than in youth, and the vital powers oscillate between health and death in much more circumscribed limits. The treatment, therefore, should be the more carefully expectant, and the less actively medicinal. Medicines also vary in effect as age advances. Salines are less suitable to the aged than the young and robust; nitre, our author thinks, should rarely or never be given, as it acts directly and injuriously upon the digestive organs of old people, inducing a kind of paralysis. The metallic remedies require to be carefully administered; arsenic is dangerous; iodine and mercury readily induce marasmus; even iron is not always safe; but zinc and antimony are indispensable. Quinine is the iron of the aged. The balsams and gums are the most generally applicable to senile diseases. In all blenorrheal affections, in nervous disorders of the thoracic and abdominal nervous systems, and in infarction of the portal system, they are particularly useful.

The approaches of disease are generally insidious in old people. Common sensation is often so dull, that the usual warnings of pain or uneasiness are not given, and impending death first excites the suspicion of disease. This insidious approach is frequently observed in the pneumonia of the aged. Partial anæsthesia of the vagus is present, increasing *pari passu* with the inflammation, and there is little or no *feeling* of dyspnea, or much febrile reaction, even when pulmonary disorganization is far advanced. Hence the necessity of numbering the motions of the thorax in all cases of slightly increased cough of old people, and especially in cold or changeable weather. For a similar reason, the premonitory signs of apoplexy, of softening of the brain, of glaucoma, and of atrophy of the alimentary canal, seldom attract due attention.

Acute diseases in old people rarely terminate by perfect or sudden crises. The excreting organs are usually impaired in both structure and function, especially the skin and kidneys. The *lysis* or solution of a disease in this mode is often slow, and more or less incomplete; and the strength of the patient is only weakened by attempts to accelerate it. It is of more importance to ascertain the relations of present disease to past pathological changes. The physician, if not acquainted with these, should extract from the patient a pathological history of his life; and thus not only a knowledge of his idiosyncrasy or constitution would be acquired, but the physician would also learn whether the symptoms were those of a primary or secondary affection, the seeds of which were sown in earlier years. The effects of residence in a tropical climate are sometimes shown for the first time in advanced years. The peculiarities, habits, and pursuits of the patient during his past life are also important to be known in all their details. An intermittent pulse is often the normal state in old persons. A regular pulse in such indicates febrile action. Dr. Canstatt writes strongly against the "inflammation-fanaticism" (as he terms it), and its influence upon the well-being of the aged. The horrid depletion practised by those physicians whose minds are impregnated with it might well make the judicious and thoughtful physician shudder. Topical bleedings are most effectual, especially if a tonic general treatment be combined. Venesection is by no means a safe operation when performed on the aged; it has been followed by immediate death; indeed Dr. Canstatt advises that the patient take a mouthful of good wine before the vein is opened.

Our author has a chapter headed with the motto "Senes bis pueri," in which an analogy is made out between the diseases of old age and infancy. Its contents are of no practical value. We express the same opinion respecting the first chapter of the second part, in which the normal conditions of lower animals is traced through the retrocedent development of old age. The arcus senilis is thus shown to be an approach to the bony rings of the sclerotica in birds; the skin, dry and scaly, resembles that of many animals, as the pachyderms; the contracted stomach of the aged is analogous to the stomach of reptiles and insects and so on, to the end of the chapter. It is impossible to divine the utility of all this; we suppose, since Dr. Canstatt says it is of little practical value, that the chapter is written exclusively for the German intellectual market. The remaining seventeen chapters are devoted to a general consideration of the pathology and therapeutics of senile diseases. The whole presents a compact view of the latter, but we must refer the reader to the work itself for details. We give the following extract as an example. It is our author's description of the phenomena of inflammation in aged people, termed by Autenrieth *neuroparalytic*, by Schönlein *neurophlogistic*:

"*a.* The volume of the inflamed organ is increased, and its absolute weight augmented; whether its density and specific gravity be increased also is doubtful.

"*b.* The large vessels, particularly the veins near the inflamed organ and in its parenchyma, are dilated and filled with dark blood. This thick congeries of vessels abounding with dark often blueish blood, is particularly distinct in arthritic ophthalmia.

"*c.* The redness of the inflamed organ is dark, violet, or almost like a cherry brown, and of a dirty hue; it is not so equally diffused as in younger subjects, but more dispersed, radiated, or punctated. The vascular tissue is not so thick, and the overfilled vessels are more distant and isolated.

"*d.* In inflammation of old people the affected tissue is softened, friable, pulpy, and easily torn with the finger.

"*e.* Transparent structures become opaque, even with a slight degree of inflammation. . . . . . . . . . . . .

"*f.* The products of inflammation are frequently found after death, as mucus in the bronchi after bronchitis; water in the serous ones, hepatization of the lungs, &c.

"*g.* The arterial system has much less share in the inflammatory action than in young persons, and new vessels are formed in the inflamed structure with much less facility. The *zoogen* is more unpliant and impenetrable. . . . . . . . . . . . . . . . . .

"*h.* Consequently the retardation of the venous circulation in the inflamed structure is remarkable; this constitutes the main characteristic of inflammation in old age. They deserve, *κατ' εξοχην*, the name of venous inflammation." . . .

"*k.* With respect to the heat of inflamed parts, either it is small in amount or is prickly and unpleasant. The more distant parts are often cold; in the inflamed parts themselves the patient experiences an intolerable heat.

"*l.* The *turgor vitalis* is generally diminished, and even in the inflamed structures is not increased.

"*m.* The functions of the inflamed organ are half paralysed, especially where nervous influence is necessary to their performance. If a secreting organ be inflamed, as, for example, the pulmonary mucous membrane, the mucous secretion itself is increased, but the bronchi themselves are half paralysed, and are only partially capable of expelling the increased mucus. The same occurs in inflammation of the bladder. Since the sensibility of old persons is obtunded, their inflammatory affections are seldom accompanied by the severe pain observed in young patients, but are the more dangerous and mischievous on this account, because pain is the sentinel of health, and its intensity affords a measure of the intensity of inflammation. . . . . . . . . . . . . . .

"*n.* As a consequence of this sub-paralytic state, the symptoms of inflammation are often intermittent in old people. Their nervous irritability requires longer intervals to recover itself. This intermission or remission of the symptoms of reaction is no proof of a less degree of disease, but rather a forerunner of complete paralysis. Consequently, senile inflammatory diseases are distinguished by a tendency to paralysis, or by terminations which are the results of paralysis. Sometimes they consist solely in paralysis, or in paralysis of the nerves and vessels conjoined, (gangrene, softening, paralytic effusions.)

"*o.* The general health is affected at a much earlier period in the inflammatory affections of the aged than in those of the young. The prostration of strength is remarkable, the patient feels singularly week and feeble." (pp. 197-200.)

In senile hectic fever, Dr. Canstatt recommends the following as particularly useful: Phosphorus, gr. iv, sulphuric naphtha ʒj; Mix; ten drops to be taken every two hours in a little water. The extr. Cardui Benedicti is almost a specific (he states) in chronic catarrh complicated with debility of the digestive organs.

In the sixteenth chapter, Dr. Canstatt treats of scrofula, gout, infarctions, lithiasis, and ossification, as being closely related, especially gout and scrofula, and gout and lithiasis. There are several interesting points developed in this chapter, but as we could only discuss them critically, and for this have no space, we pass on to other matters. The whole chapter, whether considered theoretically or practically, is worthy a careful perusal.

The second volume is devoted solely to the special pathology and treatment of the diseases of the involution-period. They are arranged under five heads as they affect the brain, thoracic viscera, abdominal viscera, genito-urinary organs, and cutaneous structures. Some of these we shall notice.

*Hyperemia of the brain.* This affection deserves special attention, because it is generally the precursor of other and more serious diseases of the encephalon. It is usually connected with partial obliteration of the capillaries, ossification of the larger arterial trunks, and varicose dilatation of the veins, and with obstruction in the return of the blood to the heart. The imperfectly oxygenized carbonaceous blood itself is a cause also of congestion. Vertigo, stupor, sleeplessness, and a predisposition to a soporose state, result from these causes, and at last end in apoplexy, softening of the brain, and dyscrasic inflammation, giving rise to paralysis.

Hyperemia may also take place solely as the result of the physical law of gravity. If aged people lie long in the same position, the slowly circulating blood sinks to the most dependent point, and accumulates in the cerebellum, and sopor, paralysis, &c. follow; just as hydrostatic pneumonia is developed in aged individuals who lie long on the back; and hence the practical maxim never to allow persons of this kind to rest long in the same position, especially in long-protracted diseases.

"*Cerebral hyperemia* is not always characterized by distinct symptoms. It may occur in a slight degree, and during relatively good health. Persons so affected complain of headach, dizziness, dulness, and stupor, or wakefulness; they feel weak, indolent in spite of themselves, and have a heaviness in their limbs. The pulse is sometimes full and tense, the face frequently red, particularly after lying down, or during sleep, or on awaking from sleep; the jugular veins are turgid and undulate; the venous network of the face is dilated and full; even the veins of the conjunctiva are varicose and prominent. Formication and titillation of the extremities are soon added to these symptoms. They often disappear entirely for a time, and then return again in all their intensity, especially after eating to repletion, or after muscular efforts, straining at stool, &c. Apoplectic attacks, with or without sanguineous effusion, may suddenly supervene; or even paralysis, for which no other cause may be found after death than simple hyperemia. The symptoms may differ according as one or other part of the brain is hyperemious." (Vol. ii, p. 3.)

Hyperemia of the brain, as Dr. Canstatt truly remarks, is rather a morbid predisposition than a specific disease. It is usually observed in the plethoric, with abdominal turgescence. On the other hand, individuals predisposed to softening of the brain are weak, have a cachectic bloodless look, and a small pulse. If individuals of the former constitution complain of flashes before the eyes, noises in the ears, difficulty in swallowing or speaking, stiffness and contraction of the muscles of the neck, restlessness during sleep, &c., in addition to the other symptoms of hyperemia, an apoplectic attack will quickly follow unless immediate relief be afforded.

The treatment of hyperemia must have direct reference to its causes. Sometimes, however, these are not apparent, and very often the highest diagnostic skill can scarcely decide whether the symptoms complained of are those of active hyperemia or of debility. If plainly of the former, venesection is necessary; if doubtful, a tentative treatment should be adopted; laxatives and gentle diuretics, with leeches to the anus or cupping of the back and neck.

We need not follow Dr. Canstatt through his notice of vertigo, coma, agrypnia, and syncope, considered as distinct diseases. They often depend upon the same causes as hyperemia, or on others well known, doubtless, to our readers. Agrypnia is sometimes an urgent symptom, as the patient becomes much debilitated by want of sleep. It sometimes depends upon dietetic errors with regard to time, or quality, or quantity. These should be pointed out. Musk is useful in cases dependent upon psychical irritation; opium is dangerous; hyoscyamus is better, or lactucarium (from gr. j to gr. ij); belladonna is recommended by Jahn; but this we should think quite as dangerous as opium. A pillow stuffed with hops has been found useful.

Syncope in old people, Dr. Canstatt thinks, is undoubtedly of cerebral origin, and always a serious symptom. In its highest degree it borders on the so-called nervous apoplexy. Our own experience would lead us to say that syncope is rare in old people, except as premonitory of paroxysmal diseases. When a symptom of disease of the heart follows uterine hemorrhages, it is of course an important one.

*Cerebral apoplexy.* This disease is treated of at great length and with much minuteness of detail. Dr. Canstatt presents a very complete view of the affection in all its forms. He divides it into three stages, 1, of congestion, or molimen hæmorrhagicum; 2, of paralysis; 3, of reaction or resolution. The symptoms of the first stage are mainly those of hyperemia already detailed. These may continue for months or years, and present oscillations in intensity, for the most part dependent, Dr. Canstatt thinks, on variation in atmospheric density and pressure. Sometimes attacks of simple congestive apoplexy will precede the more serious attack consequent on sanguineous effusion. The former is the *coup de sang* of the French, and is known from the latter principally by this, that all the extremities are usually affected. There may be hemiplegia or partial paralysis, however, as happens to those epileptics who remain hemiplegic for a day or two after a succession of fits. This form may be periodic. Rüsch relates an instance in which an individual, aged sixty-five, experienced seven paroxysms at intervals of one month between each, without suffering any bad consequences. (Mediz. Annalen, Bd. i, Heft 3.)

The second stage of apoplexy is that which gives the name to the disease, constituting emphatically "the fit." The third stage commences with a return of sensation and motion to the parts which had lost them; this return being dependent upon the reactive energy of the brain itself, and not on the absorption of the extravasated blood. This reaction is displayed in the lower extremities sooner than in the upper, and power returns in the tongue and muscles of the face before it is felt either in the arm or leg. Sometimes there are reflex movements; the muscles of the leg or the side of the face affected being convulsed without consciousness. Sooner or later vascular reaction comes on, and there are febrile exacerbations, flushings of the face, headach, and occasionally epistaxis; the pulse rises, the skin is hotter, the urine high coloured. Chronic paralysis is the termination of this stage.

Dr. Canstatt devotes considerable space to the diagnosis of apoplexy. He classes the symptoms as they are connected with the motive, sensitive, sensual, and intellectual powers. Apoplectic paralysis of the face is distinguished from rheumatic paralysis, and from that dependent on granular softening or on nervous apoplexy, or on serous apoplexy (hydrocephalus) or on disease of the spinal cord. The pathological anatomy of apoplexy has been so often and so well described, that Dr. Canstatt could scarcely add much to it. It appears, however, from Fuchs' researches,* that the softening of the brain usually found round the clot is characterized by peculiar symptoms during life. Where softening follows effusion, the pulse becomes small, weak, quick, and irregular; the consciousness after the first attack is imperfect, a comatose state is induced, and another fit follows, although there is no fresh effusion. The patient usually dies of a slow fever. Fuchs found this secondary softening in almost all cases of apoplexy in which the patient died in four, seven, or ten days without suffering a fresh attack.

Apoplexy is the disease most fatal to persons in the decline of life. Walther, however, places the number too high when he asserts that nine out of ten die of apoplexy. Rochoux states that of 69 cases of apoplexy, 49 occurred in persons aged from fifty to eighty, and 37, or more than one half, in those from fifty to seventy years of age. Men are more subject to it than women. Of 2297 cases collected by Falret, 1670 were men. According to P. Frank, 637 men and 604 women died of apoplexy in the hospital at Vienna, between the years 1784 and 1804.

Dr. Canstatt thinks too much stress has been laid on the connexion between apoplexy and cardiac diseases. The changes in the vascular system which predispose to the latter, predispose also to the former; the two diseases are rather coincident than casual. The gouty constitution predisposes. "Arthritis quæ quibusdam calculosam," says Stoll, "aliis vero apoplecticam dispositionem inducit." Atheromatous disease of arteries is an usual concomitant of gout, and gouty inflammation of the meninges may readily induce congestion and rupture of these brittle atheromatous vessels. Atmospheric changes so constantly induce apoplexy, that the disease has been observed to be epidemic, as stated by Baglivi and others. Hippocrates observed that cases were more frequent in winter, and Andral's statement, that of 177 cases, 60 were in winter, 42 in spring, 35 in summer, and 40 in autumn, confirms the observation. The secular divisions of the seasons is unsuitable to medical statistics. The vernal equinox is not physiologically the commencement of spring, but the middle; as the winter solstice is the middle and not the commencement of winter.

Falret attempted to show that certain epochs are more favorable than others to apoplexy, as follows. In the ten years

| | |
|---|---|
| From 1794 to 1804 there were ............ | 339 cases. |
| From 1804 to 1814 ............................ | 997 ... |
| From 1814 to 1824 ............................ | 919 ... |
| | 2255 |

Very little reliance can be placed on statistics of this kind, for obvious reasons.

* Beobach. und Bemarkung. über Gehirnerweichung.—Leipzic, 1838.

Our author next presents us with a good view of the treatment of apoplexy, but little that is new to the English reader. He recommends bleeding at the equinoxes to those predisposed to the disease. Bleeding from the arm is in all cases preferable to section of the jugular vein.

*Encephalomalacia senilis,* or softening of the brain, in old age. A very complete history of this affection is given; its course, complications, diagnosis, and pathological anatomy being systematically described, and reference continually made to the most recent authors on the subject, particularly to the work of Fuchs before quoted. This disease we have reviewed very recently.

Meningitis, acute and chronic hydrocephalus, atrophy or senile cretinism, and the pseudoplasmata of the brain, are considered in succession. Under the last head are included tubercular and cancerous degenerations, aneurisms, and hydatid and other tumours, as they affect the aged.

*Pneumonia senilis.* This is the first disease treated of in the second division. It occurs in two forms: in the one its attack is sudden, and its symptoms unmistakeable; the other proceeds more slowly and mischievously, and often the true nature of the disease is so concealed that the patient appears quite well, attends to his usual business, goes about, and eats with appetite, until at last he feels exhausted, and dies quite unexpectedly. Not unfrequently in such cases a large portion of the lungs is found after death in a state of suppuration. The following is our author's description:

"When attacked with pneumonia, old persons rarely complain of dyspnea. The breathing is nevertheless frequent and difficult, the chest is more energetically moved than usual; the patient lies motionless on his back, and is unable to turn on his side; he speaks with difficulty, and coughs between almost every word. The physician should never neglect to count the number of respirations in those old persons who complain of slight oppression or stuffing of the chest. The pneumonia of old people is also rarely accompanied by pain; nothing more is commonly complained of than an indistinct uneasiness which seldom becomes fixed. The patient is often deceived as to the seat of pain, and refers it to a part of the lungs quite free from inflammation. The absence of pain must not lead the physician to suppose that the inflammatory action is slight, for even when a large part of the pleura is inflamed, the pleuritic stitch is often wanting. Pneumonia in old subjects often begins with arthritic symptoms, and the characteristic signs of the disease appear only after venesection. There is generally a cough, but it is so slight as not to be noticed by the patient. In many cases it does not differ from the cough of bronchitis; but it generally consists in one or more little convulsive *puffs* without antecedent inspirations; it may, however, be exceedingly troublesome. It is sometimes a rattling, sometimes a gurgling cough. Where there is no blood in the sputa, they are grayish or greenish, and opaque, seldom transparent and tenacious; at an advanced age they are puriform. They are, however, more or less tinged with blood, and are sometimes almost altogether sanguineous; at a later period this goes off, and the sputa are like chocolate or plum-sauce. An habitual bronchorrhea sometimes modifies the sputa of pneumonia, and sometimes in those old patients who have been addicted to spirit drinking, the breath has a disagreeable putrescent smell, even when there are otherwise no suspicious symptoms. The physician will therefore be careful not to infer from this symptom that there is gangrene of the lungs." (pp. 93-4.)

This odour, we would observe, is not gangrenous but fecal. No physician who has observed cases of this kind can mistake the one for the other. The fecal odour is by no means confined to old people, nor to pneumonia; it is rather an accompaniment of some curious forms of bronchitis.

On noticing the physical signs, Dr. Canstatt describes the modification induced in them by the physiological peculiarity of the aged. The chest generally is more sonorous, but at the inner half of the clavicular region is often less resonant on account of the black or gray induration so common in the apices of the lungs of old people, and in old females by the peculiar bowing of the clavicles. The respiratory murmur is more vesicular and clearer, and resembles rather the murmur of bronchial respiration.

Delirium is commonly one of the rational signs of pneumonia, and the patient complains more frequently of pain in the head than in the chest. The face is red and often livid or earth-coloured. The nostrils are dry, the tongue dry, white, crumpled up, and often dark and covered with a sooty fur. Other symptoms are enumerated, and it is finally stated and truly, (as we ourselves know,) that all the characteristic symptoms may be absent when the disease is intense. This form is most frequently seen in persons with structural disease of the heart.

Pneumonia is an acute disease in the aged. Hourmann and Dechambre found the average duration in 33 cured cases to be 14 days; in 76 it was fatal in 7 days.

*Treatment of pneumonia.* If the inflammation be peracute, bleeding should not be neglected, however old the patent may be. Octogenarians will often bear it well. The pulse is, however, no criterion for the operation or for its repetition; spare old men bear it better than corpulent subjects in most cases. Local bleedings had better follow the general.

If the hepatization has passed into suppuration, bleeding is dangerous, expectoration is prevented by the debility induced, and the patient may die suffocated a few hours after.

As to the medicines, Dr. Canstatt recommends us to administer sal ammoniac, (the hydro-chlorate of ammonia,) in preference to nitre. It diminishes vascular action, and assists expectoration without debilitating. Large doses of tartar emetic may be useful in young people, but are dangerous in the old, inducing colliquative diarrhea and general prostration. The white oxide of antimony in large doses, as recommended by Recamier is better. Senega and squills are useful when the expectoration is stopped, or the strength failing. Flowers of benzoin, (a good old remedy,) were formerly given in such cases, gr. ij to iv, every two hours. Narcotics are of doubtful utility, and diffusible stimulants should only be administered in cases of prostration.

The remaining diseases of the lungs treated of are, bronchitis, chronic pulmonary blenorrhea, moist asthma or dilatation of the bronchi, chronic croup in the aged, asthma in all its forms, hydrothorax, phthisis, hemoptoë and pulmonary lithiasis. Our crowded volume will not permit us to notice in detail either these or the other affections of the heart, abdominal viscera and external surface. The student of German literature will do well to peruse the work for himself. There are some forms of disease, however, dependent, according to our author, upon the gouty diathesis or upon imperfect urinary secretion, which we will glance at, as we think his views will interest many of our readers.

We have already remarked on the connexion between gout and apoplexy. Arthritic tubercular disease may be developed in the brain. In these cases, paroxysms of cephalæa resembling clavus, take the place of the regular fit, and the pain, gradually increasing, may become so intense as to deprive the patient of sleep and rest. If the disease continues, structural changes take place, and periodical epilepsy or convulsions follow. These, in their turn, may exhibit the type of arthritis, or may be more severe at the periods when gouty paroxysm usually come on. They are generally unaccompanied by an aura, and are most frequently hemiplegic, and terminate either in apoplexy, paralysis, or idiocy.

*Chronic coughs* are often gouty. In these cases the paroxysms of coughing come on most frequently about midnight, or two o'clock in the morning. Occasionally, a bronchitic affection alternates with fits of the gout. *Asthmatic* affections are often complicated with the latter. Three species of asthma are specified by our author, which we think are so closely allied as to constitute only varieties of the same disease. These are asthma *urinosum*, *arthriticum*, and *gonorrhoicum*. The last mentioned is named by Schönlein, and described as follows: Long after the suppression of a gonorrheal discharge, (say, from 15 to 20 years!) slight oppression of the chest, and dyspnea are felt, especially after any exertion, which at first soon disappear, and are scarcely noticed by the patient until after some exciting cause, as, for instance, excess of wine, or strong emotion, a perfect asthmatic paroxysm comes on. The cough is at first dry, but subsequently blood and masses of coagulable lymph, varying in size from that of a hazel-nut to an apple, are expectorated, or thrown off by vomiting. Unless this takes place the patient dies during the fit, suffocated. *Arthritic* asthma appears in gouty patients, and the paroxysm is preceded by the premonitory signs of a fit of gout, as uneasiness in the precordial region, flatulence, acid eructations, cramps of the legs, loaded urine, &c.; but instead of the usual fit, a paroxysm of asthma comes on about midnight, and is distinguished from others by the great irritation of the pulmonary mucous membrane. In consequence of this the countenance is remarkably livid, the jugular veins distended, bloody foam comes from the mouth, and towards morning, when the fit is remitting, large masses of mucus mixed with blood are vomited or expectorated. The paroxysms, as to their duration, return, and critical terminations, correspond in all points to paroxysms of gout. The *asthma urinosum* closely resembles the arthritic as to its mode of attack and termination; but there are some other general symptoms peculiar to it which we shall subsequently notice, and the expectoration is more glairy, tenacious, and has often a saline taste and urinous odour. Both these forms of asthma readily pass into apoplexy.

The treatment of these affections is not very diverse. The asthma gonorrhoicum, we believe to be an imaginary disease so far as regards its cause; the affection described under that name being a variety of the arthritic or urinous form; it is best treated by emetics. In arthritic asthma, the symptoms are so urgent that, besides the general treatment, bleeding to eight or twelve ounces is almost indispensable to prevent suffocation. Musk is also of the greatest use as a palliative. In the urinous asthma, bleeding is rarely necessary; diuretics should be administered, but very carefully as renal disease is often coexistent, and the irritation of the kidneys may readily pass into inflammation.

*Arthritic hydrothorax* is usually developed between the ages of forty-five and sixty. Men of strong constitutions, and great eaters whose assimilative powers are weakened by a sedentary life or mental labour, are most usually the subjects of it. It is sometimes acute, and then is the consequence of retrocedent gout. When it is chronic, the pectoral symptoms are preceded by the visceral derangements usually premonitory of a gouty fit, but instead of pain in an extremity, dyspnea, palpitation, intermittent pulse, &c., suddenly come on. In a while, these become less intense, but do not altogether disappear, and are periodically aggravated. Structural disease of the heart and large vessels is the consequence of these attacks. It is doubtless in cases of this kind, (although the opinion is not noticed by our author,) that a solution of the disease has been observed to follow on some critical profluvium, as profuse sweats, and urinary discharge. We should also be inclined to class the *intermittent phthisis* of the involution-period, particularly of its commencement, with these affections. This thoracic affection is apparently dependent upon peculiar ulcers of the legs, which we shall notice presently; as these heal or discharge less, the pectoral symptoms appear. The latter occur specially in autumn, and are accompanied by symptoms of fever; but the bowels are confined, the urine scanty, dark, saturated with saline matters, and throwing down a copious precipitate. It appears to us that the disease is really a constitutional peripneumonia ending in suppuration, and that the ulcers only act as outlets for the materies morbi, and are analogous to issues. At all events we have witnessed cases of pulmonary diseases in elderly people of decided gouty constitution which were manifestly of this kind. The pulmonary affections connected with hemorrhoids or fistulæ ani are also allied to this. The *lithiasis pulmonum* is usually of gouty origin, and analogous to the lithiasis of the arteries. The concretions are deposited in the serous or subserous cellular tissue of the bronchial ramifications, or of filled-up tubercular cavities, and constitute a sort of imperfect ossification. They may attain a considerable size, and are composed of the phosphate and carbonate of lime, phosphate of magnesia, some cholesterine, oxide of iron, and silica.

Structural changes and diseases of the heart and its appendages in old people, are eminently connected with the gouty or rheumatic constitution. At first, they often alternate with imperfect fits of the gout, until the latter entirely disappear. The connexion between the gout and the renal secretion is also manifest in these cases; the structural change in the heart, says our author, scarcely manifests any of its peculiar symptoms so long as the kidneys perform their function properly. The more scanty and imperfect the urinary secretion, the more urgent the symptoms of cardiac disease.

We need not go through the individual symptoms of cardiac disease which are dependent on arthritic metastasis. Of course, there may be gouty palpitations, carditis in all its forms, cardiostenosis, or valvular disease, angina pectoris, &c., and these may be either acute, peracute, or chronic. One or other of these arthritic cardiac affections is so frequently observed in old people that Frank pronounced the gouty diathesis to be a *senectus anticipata.* As regards the renal function, this is highly probable.

*Arthritic affection of the stomach.* It is the humoral pathology that illuminates most clearly the diseases of old age, although so constantly connected with structural change. Impaired function of an important excretory organ, as the kidneys, is necessarily followed by impaired functions in other organs. The matter which ought to have been eliminated, becomes a true *materies morbi*, circulated as a foreign body through the system, and is deposited abnormally on structures otherwise healthy. The kidneys have both an anatomical and a physiological connexion with the testes, and it is more than probable that the impaired function of the former organs is closely connected with the impaired function or morbid reaction of the latter. Be this as it may, the materies morbi which determines arthritic metastasis operates when located on the stomach as a most active and dangerous poison. We know of no disease more painful, and requiring more anxious care and prompt treatment than the arthritic gastritis of old men, or gout in the stomach as it is popularly termed. In all cases of this kind, the practitioner must establish a decided diagnosis at once, and unflinchingly act upon it. Delay is emphatically dangerous here, as disorganization and perforation of the stomach very quickly succeed to the arthritic inflammation. Large doses of morphia and diffusible stimulants are the most effectual remedies at the outset, combined with derivatives to the extremities. When, however, the inflammation is established, and all remedies are immediately rejected, we have found great benefit from a very copious enema, (say a gallon,) of warm water with from half an ounce to an ounce of sulphuric ether, and three or four drachms of laudanum. The cæcum, and the ureters and the urinary bladder, but especially the cæcum, are also the seat of this arthritic inflammation, a fact not noticed by Dr. Canstatt.

*Ascites venosus s. periodicus.* We extract Dr. Canstatt's description of this affection entire:

"This disease attacks only old persons, sixty and seventy years old, who at an earlier age suffered from regular or irregular gout, hemorrhoidal congestions, or hemorrhoids. Although these pathological excretions have ceased, their existence in the system is still manifested in cold, damp weather by venous turgescence in general, and particularly of the portal system, by oppression in the hypochondria and hypogastrium, and tension of those regions; by fulness and undulation of the external veins, as for example, those of the neck and of the hemorrhoidal veins. There is, however, no hemorrhoidal discharge; the general reaction by which the *materies morbi* was formerly eliminated is wanting, and, falling short of this, excites only febrile irritation, with some degree of cutaneous itching and efflorescence, which disappear after a sweat. The venous congestion and the want of reaction is followed by external and internal effusion and dropsical swelling. The surface becomes œdematous, and, what is remarkable, the higher parts first, namely, the genitals, nates, loins, and hips: from these the œdema descends to the legs. The patient passes a small quantity of urine, the colour of brick-dust, and throwing down a precipitate a finger thick, containing uric, purpuric, and erythric acids; and now the abdomen enlarges. In the beginning the œdema is not permanent, but continues a few days and then disappears, the patient passing more urine and perspiring; ultimately, however, it becomes permanent.

"In the commencement of the affection these symptoms continue as long as the hemorrhoidal or gouty paroxysm formerly continued, namely, from one to four weeks, and terminates with a critical sweat, or a copious urinous deposit; the abdomen then collapses, and the patient remains free from the disease until some particular influence—the seasonal changes or the accumulation of *materies morbi* —excites a fresh paroxysm. It is on this account that the disease has been termed *ascites periodicus*.

"This form of abdominal dropsies is an anomalous form of gout, developed partly in consequence of senile debility, partly by debilitating external influences. . . . . .

Anomalous arthritis sometimes alternates with venous ascites, sometimes with arthritic hydrothorax and asthma. This wandering form is the more dangerous, as it quickly gives rise to those structural changes in the heart and lungs which of necessity end fatally." (pp. 317-18.)

Dr. Canstatt recommends leeches to the anus, hypochondrium, and hypogastrium, and cupping along the spine. Small doses of chelidonia and aloes may excite a hemorrhoidal flux. To remove the effusion, cathartics should be administered two or three times a week in conjunction with diuretics and diaphoretics. In the arthritic form, the drastic purges are dangerous. The kidneys and skin should be acted on rather than the bowels. Sulphureous preparations, Dover's powder, acetate of soda, aconitum, rhododendron, guaiacum, and tincture of nicotiana topically are the remedies recommended. We pass over the arthritic affections of the kidneys, bladder, and ureters, to a form of general disease occurring now and then in aged persons, termed by our author and other German writers,—

*Anuria seu Urodialysis senum.* This affection has been illustrated and considered systematically by Autenrieth, Schönlein, and Jahn. It consists in an almost total suppression of urine, not occurring suddenly but gradually, so that the system, in becoming charged with the urinary secretion, becomes also accustomed in some degree to its presence, and an imperfectly vicarious excretion takes place from different organs. The skin is dry, the urine small in quantity and scalding, and there is frequent micturition. The digestive organs are deranged, the tongue covered with a white fur, and the rest broken by the frequent calls to pass urine. Urodialysis assumes various forms.

"*a.* It sometimes appears as *rheumatismus urinosus*. The patient complains of irritating pain in the limbs, exacerbated at night. For the most part they follow the course of the sciatic nerve and are periodic.

"*b.* The skin is most frequently the seat of urodialytic irritations.

" Sometimes it appears as an intolerable itching, aggravated at night, and depriving the patient of sleep. The skin is often rough or covered with a chronic eruption in the form of papulæ or clusters of small vesicles, from which, however, the head and face are free. The patient scratches the heads off these and they are then covered with a small bloody scab forming *prurigo* and *epinyctis senilis*. Sometimes these vesicles are larger and belong to the pemphigus class. As regards the face, *herpes exedens* may attack the lips, cheeks, and eyelids; it begins with a hard, livid, little knot, which, when scratched off, leaves a small jagged ulcer that secretes a thin ichor, is covered with a crust, and continually gets deeper and deeper. Sometimes there is cancer of the tongue.

" As regards the lower extremities, the urodialytic irritation gives origin to the formation of chronic ulcers, the so-called " salt fluxes;" these are commonly situate on the anterior and inner surface of the leg, and are first made by a scratch or slight injury to the skin. They are painful, readily extend, are flat, have hard but not raised edges, and secrete an acid, acrimonious thin ichor, very nauseous and sometimes of a urinous smell, which when inspissated on the ulcer, forms a thin black crust.

"*c.* The conjunctiva is also sometimes the seat of an irritating discharge; it becomes red, relaxed, and pours out an acrid mucus which corrodes the eyelids, changes the adjoining cutaneous structures into mucous structures, destroys the eyelashes and commissures of the eyelids, renders the lucid cornea opaque, excoriates the cheeks, and brings on ectropria, trichiasis, and nebulæ. This affection is known as *lippitudo senilis*.

"*d.* Urodialysis may excite numerous forms of disease in the thoracic viscera. Many of these patients suffer from constant dyspnea, short and anxious breathing, and palpitation.

"The *asthma urinosum* has been already described. Urinary acrimonies also excite those forms of chronic pneumonia and periodical phthisis previously alluded to. Hydrothorax often quickly follows either on the asthmatic affection or on obstruction of the discharge from the urinary ulcers in the extremities.

"*e.* As regards the brain, apoplexy is the sequel. The patient complains of vertigo, headach, heaviness and dulness in the head. An acute burning sensation in the nucha and occiput is a certain premonitory sign of urinous apoplexy. A subacute typhoid state may also be developed, with stupor, coma, passive delirium, and putrid scorbutic symptoms." (pp. 385 7.)

Of course several of these forms of disease may appear simultaneously; prurigo, for example, usually accompanies urinous asthma. Death results either from apoplexy or thoracic disease, or the patient is worn out by the intolerable itching. The sufferers are almost invariably above sixty, and principally in the lower classes of society. In fact it is usually a disease of decrepitude and cutaneous uncleanness.

The remaining arthritic disease of old age are in the fifth division of Dr. Canstatt's work. Of these *prurigo vulvæ* and *prurigo podicis*, as well as prurigo generally, are allied to urodialytic prurigo. It must be observed, however, that in arthritic prurigo, the irritation most usually attacks those portions of the surface connected specially with the genito-urinary system, as the chest, shoulders, axillæ, and the genital organs; the inner surface of the vagina, according to our observations, is as frequently the seat of prurigo as the outer. *Gangrena senilis* is sometimes distinctly connected with anomalous arthritic affections. Morgagni quotes three remarkable cases of this kind, in which the cure of gangrenous ulcerations of the heel were preceded by profuse critical discharge of urine, and death resulted from inflammation of the bladder.

*Erysipelas senile.* This is an affection the practitioner should thoroughly understand, as it differs altogether from the common erysipelas of surgical writers, as well in its course and causes as its treatment; it is in fact cutaneous gout. This erysipelas is usually seated in the lower extremities, although it may be erratic, that is to say, migrate to other parts, just as arthritic affections in elderly people will. There is usually a livid red spot first seen on the anterior surface of the tibia more or less circumscribed, and which usually disappears on pressure. The general symptoms are those of the arthritic or hemorrhoidal diathesis; as tension of the epigastric or hypochondriac regions, constipation, anorexia, bitter taste in the mouth, headach. Indeed, in many cases there are first regular fits of gout, then hemorrhoidal discharges; afterwards these become irregular and anomalous, and then there are periodic attacks of the erisipelas. There are very rarely vesicles in this form of erysipelas not even when gangrene comes on, but the affected limb is often œdematous, and the veins varicose.

This erysipelatous inflammation, like the gouty, is of a critical kind; it is a sign that the blood is undergoing a purifying process, and cannot be repelled without danger. It exhibits weakness of the system, and therefore in conducting the treatment, depleting remedies should be avoided. Such are very successful in causing the inflammation to disappear most quickly, but it is because the skin ceases to be the organ of the crisis. We have seen the brain and stomach metastatically affected in consequence of the supposed cure of the disease. Females are more liable to it than males, and as it often appears in them at the cessation of the menstrual discharge, it has been termed *erysipelas climactericum.*

In treating senile erysipelas the usual local applications should be carefully avoided. There is no room for trifling. Structural disease of the brain, or of the thoracic or abdominal viscera, will quickly follow its repression. The patient must be thankful that he has the disease, and diaphoretics should be given, as Dover's powder, valerian, camphor, and the acetate and succinate of ammonia, to encourage the cutaneous action. If gastric symptoms should appear, an emetic will be useful, followed by a laxative, and if the disease be periodic, an aperient should be given every four weeks. The affected limb must be wrapped in pads filled with aromatic herbs, and the inner surface rubbed with camphorated liniment. If the erysipelas have disappeared, hot fomentations, the ammoniated liniments, and blisters should be applied to the limb to recall the morbid action to the surface. When the patient has tolerably recovered from the attack, an issue should be formed at a convenient point, and carefully kept open.

Erysipelas in persons turned sixty years of age is usually a *malum signum.* It is premonitory of an approaching break-up, and of death in from one to four years, according to the strength of the individual. This usually takes place in spring or autumn, but most frequently during the latter season.

Having selected these gouty affections from the others described by our author, we must here close our review. We can only here repeat that this kind of publication was a desideratum which Dr. Canstatt's work supplies. We should be glad to see it in an English dress, with notes, as it would thus fill up a manifest gap in our own medical literature. The text, however, should be abridged and the matter somewhat rearranged, so as to avoid those many repetitions as to the exciting and predisposing causes of disease in which Dr. Canstatt indulges. They may give the work an appearance of fulness and completeness, but render it larger than necessary; no trifling fault in these book-making days.

